# Nanoarchitectonics of BN/AgNWs/Epoxy Composites with High Thermal Conductivity and Electrical Insulation

**DOI:** 10.3390/polym13244417

**Published:** 2021-12-16

**Authors:** Xue Li, Ling Weng, Hebing Wang, Xiaoming Wang

**Affiliations:** 1 College of Material Science and Engineering, Harbin University of Science and Technology, Harbin 150040, China; lxue233333@163.com (X.L.); 18745025900@163.com (H.W.); 18648280951@163.com (X.W.); 2 Key Laboratory of Engineering Dielectric and Its Application, Ministry of Education, Harbin University of Science and Technology, Harbin 150040, China

**Keywords:** epoxy resin, boron nitride, silver nanowires, thermal conductivity

## Abstract

To promote the construction of the thermal network in the epoxy resin (EP), a certain proportion of silver nanowires (AgNWs) coupled with the hexagonal boron nitride (BN) nanoplates were chosen as fillers to improve the thermal conductivity of EP resin. Before preparing the composites, BN was treated by silane coupling agent 3-aminopropyltriethoxysilane (KH550), and AgNWs was coated by dopamine hydrochloride. The BN/AgNWs/EP composites were prepared after curing, and the thermal conductivity and dielectric properties of the composites was tested. Results showed that the AgNWs and BN were uniformly dispersed in epoxy resin. It synergistically built a thermal network and greatly increased the thermal conductivity of the composites, which increased 9% after adding AgNWs. Moreover, the electrical property test showed that the addition of AgNWs had little effect on the dielectric constant and dielectric loss of the composites, indicating a rather good electrical insulation of the composites.

## 1. Introduction

Scientific development leads to higher standards for motors, electronic packaging, and so on. Technological progress has made electronic devices smaller and more efficient [1], so more heat is generated. Excessive heat generation is the cause of heat accumulation, which affects the working environment and working temperature of electronic devices. Long-term exposure to high temperature will greatly shorten the service life of electronic devices. In order to prolong the service life of an electronic device, it is necessary to improve its heat dissipation capacity and keep its working temperature within a normal range. Therefore, it is essential to study and prepare materials with high thermal conductivity to solve the problems caused by excessive heat accumulation [2,3,4]. Compared with other materials, polymer materials have better processing properties. Through structural changes, their properties can be changed to meet people’s needs. The most important thing is that polymer materials have good electrical insulation [5,6,7,8] and certain adhesion [9,10]. Therefore, they are widely used in motor, electronic packaging, LED packaging, aerospace [11,12,13,14,15,16] and other fields. However, the poor thermal conductivity of polymer materials limits its application. Therefore, it is imperative to develop polymer matrix composites with high thermal conductivity and insulation. At present, the mainstream approach is to select some inorganic nanoparticles with high thermal conductivity as fillers, which are added into polymers to prepare high thermal conductive composites. Traditional fillers mainly include metals such as Al, Ag, Cu and Mg [17,18,19] or inorganic carbon materials such as carbon black and graphene [20,21,22,23,24,25]. However, the strong conductivity of the above fillers significantly reduces the insulation of the composites, which limits the composites’ application in motors, electrical appliances and electronics.

Recently, more attention has been paid to nitride fillers, which are widely used in various fields due to their compact atomic crystal structure, excellent thermal conductivity and good electrical insulation. The versatile properties of BN make it suitable for a wide variety of applications. Mortazavi et al. used a molecular dynamics simulation to study the effect of grain size on the thermal conductivity of polycrystalline BN at different temperatures [26,27]. Polycrystalline hexagonal boron nitride exhibits a thermal conductivity comparable to that of single-crystal thin plates. Sun et al. used an infrared camera to observe the heat dissipation effect of BN [28]. It was found that the temperature reduction of the film was reduced by about 20% after the addition of BN, demonstrating the potential for heat dissipation of the BN film. By studying the spatial and spectral properties of the infrared thermal emission of the device. Barnard et al. found that BN and graphene can increase lateral heat transfer within the device [29]. Loeblein et al. found that the temperature of nanostructured foamed thermal interface materials consisting of three-dimensional carbon (3D-C) and hexagonal boron nitride (3D-BN) was reduced by 20% compared to conventional thermal interface materials, and thermal resistance was reduced by 25% [30]. Kargar et al. studied epoxy-based composites with BN and graphene as fillers [31]. It was found that, at high filler loadings, heat transfer in composites occurs primarily via the filler network.

The above research shows that the addition of BN can effectively improve the thermal conductivity of the composites, while not significantly reducing their electrical insulation properties. However, when BN is used as the heat conduction filler, its heat conduction mechanism still follows the traditional heat conduction network theory. This requires that the content of BN must reach a large proportion before a relatively complete heat conduction network can be formed in the epoxy resin, which often sacrifices the mechanical properties of the composite. Therefore, how to ensure the synergy of thermal, mechanical and electrical properties of composites at low filler content is one of the focuses in the field of thermal conductive composites, and the key lies in the construction of the thermal conductive network.

In view of the above problems, this study used epoxy resin as matrix, boron nitride and silver nanowires as composite fillers, and methyl-hexahydro phthalic anhydride and methyl nadic anhydride as curing agents to prepare boron nitride/silver nanowires/epoxy resin composites via curing reaction. The difference in mechanical properties and dielectric properties, as well as improvement of thermal conductivity of the composites, were studied by adjusting the ratio of BN and AgNWs in the composite fillers. A silane coupling agent KH550 was used as a surface treatment material to bring amino groups to the BN surface, improving the interfacial compatibility between BN and epoxy resin. Similarly, a biological reagent of PDA as surface coating material was used to coat a dense PDA layer on the surface of AgNWs to improve the interface compatibility between AgNWs and epoxy resin.

## 2. Materials and Methods

### 2.1. Materials

Hexagonal boron nitride (100 nm) was supplied by Yaotian Chemical Co., Ltd., Beijing, China. Silver nanowires (AgNWs) was supplied by Lengshi Chemical Co., Ltd., Suzhou, China. Epoxy resin (E-51) was supplied by Xingchen Chemical Co., Ltd., Shenzhen, China. Silane coupling agent (KH550) was supplied by Liangui Chemical Co., Ltd., Shenzhen, China. Dopamine hydrochloride (PDA) and trishydroxymethyl aminomethane (tris-Hcl) was supplied by Aladdin Chemical Co., Ltd., Shanghai, China. Anhydrous ethanol and Pyridine were purchased from Fuyu Chemical Co., Ltd., Fuyu, China. Methyl hexahydrophthalic anhydride (MHHPA) and Methyl nadic anhydride (MNA) were from Ruixiang Chemical Co., Ltd., Changzhou, China.

### 2.2. Composites Preparation

We put 20 g BN into 200 mL deionized water and 300 mL anhydrous ethanol solution; ultrasonic stirring was performed for one hour. Add 0.8 g KH550 slowly and stir at 80 °C for 2 h. Finally, the modified BN was obtained by suction filtration and washed and dried.

0.5 g Tris HCl and 0.22 g NaOH were dissolved in 600 mL absolute ethanol to prepare a buffer solution. 1 g AgNW and 2 g PDA were dissolved in buffer solution and stirred for 6 h at 60 °C. Wash the mixed solution to pH neutral.

**Figure 1:**
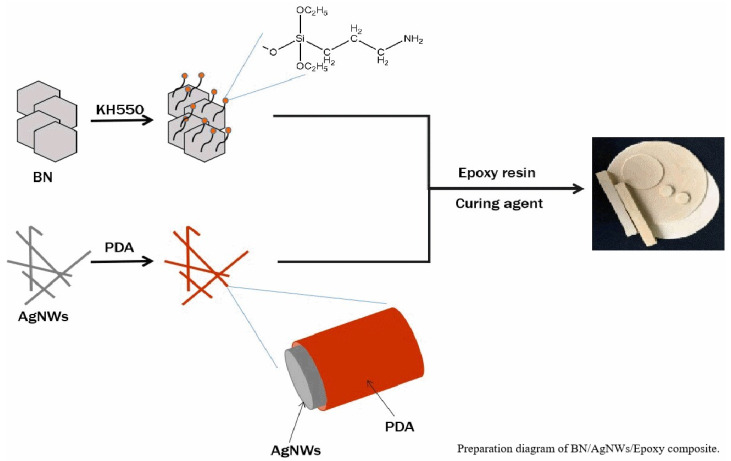
Preparation of BN/AgNWs/Epoxy composite.

Firstly, desired amount of BN and AgNWs were gradually added into the epoxy resin. For the mass ratio of AgNWs, BN in composite filler was maintained at 0:300, 1:300, 2:300, 3:300, 4:300, which was recorded as BN/Ag-0, BN/Ag-1, BN/Ag-2, BN/Ag-3, BN/Ag-4. BN fillers were added to epoxy resin at a rate of 20% by volume. Secondly, MHHPA and MNA were added into the epoxy resin as the curing agent. Vacuum at 90 °C in the vacuum drying oven for 1 h, and then adjust the temperature to 110 °C and keep it for 2 h. Finally, adjust the temperature to 180 °C, keep the temperature for 1 h, and take out after natural cooling for testing. The preparation of composites is shown in Figure 1.

### 2.3. Measurements

Infrared spectroscopic characterization of BN particles was performed on a Paragon 1000 Fourier transform infrared spectrometer (FTIR, Perkin Elmer Ltd., Akron, OH, USA). Prior to FTIR analysis, all samples were tableted with potassium bromide powder. A JSM-7500F scanning electron microscope (SEM, Japan Electronics Ltd.., Tokyo, Japan) was used to image the cross-sectional microstructures of BN/EP composites. Before being tested, the specimen needed to be quenched with liquid nitrogen. A JEM 2100F transmission electron microscope (TEM, Japan Electronics Ltd., Tokyo, Japan) was used for observing the morphologies of the BN nanosheet. The thermal analysis samples were smooth sheet-like materials with a thickness of 2–4 mm. A LFA-427 Laser thermal analyzer (Netzsch Ltd., Selb, Germany) was used to measure thermal conductivity at room temperature. Thermogravimetric analysis of epoxy composites (10 mg) was carried out using a TG-209F3 thermogravimetric analyzer (TG, Netzsch Ltd., Selb, Germany) under a nitrogen atmosphere, using a heating rate of 20 °C/min and a gas flow rate of 20 mL/min, over the temperature range of rt to 700 °C. A rectangular sample with dimensions of 40 mm × 7 mm × 1 mm (length × width × thickness) was used for measuring the dynamic mechanical properties of cured epoxy composites on an RSA-G2 dynamic thermomechanical analyzer (TA Ltd., New Castle, DE, USA). The flexural strength of composites was tested on a universal material testing machine (Shimadzu Ltd., AGS-J10, Tokyo, Japan) using a sample size of 120 mm × 10 mm × 4 mm (length × width × thickness). A layer of aluminum foil was affixed to the front and back of dry composite samples of 2 mm thickness and 30 mm diameter, with their dielectric properties and then determined using a broadband dielectric spectrometer (Novocontrol Ltd., Alpha-a, Frankfurt, Germany). The electrical resistivity of the cured epoxy composite samples (100 mm diameter × 1 mm thickness) was tested using a ZC-36 Megohmmeter (Shanghai Fine Science Instrument Co., Ltd., Shanghai, China) at 25 °C.

## 3. Results

### 3.1. Surface Treatment and Characterization of AgNWs

**Figure 2:**
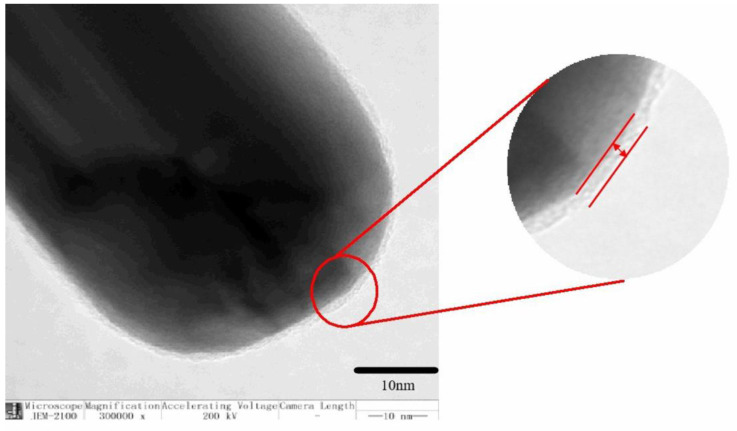
TEM of Ag nanowire coated by polydopamine.

Figure 2 shows a transmission electron microscopy of AgNWs coated with PDA. Dopamine is uniformly coated with a layer of polydopamine on the surface of AgNWs by self-polymerization in alkaline conditions. The coating of organic substance on inorganic AgNWs improves their interfacial bonding with EP.

### 3.2. Surface Treatment and Characterization of BN

**Figure 3:**
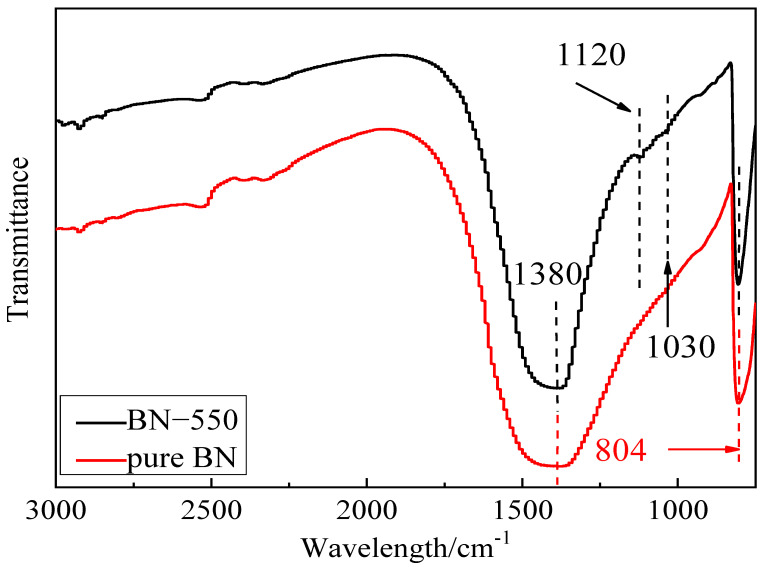
FT-IR spectra of pure BN and BN-550.

The infrared spectrum of BN filler after surface modification with silane coupling agent KH550 is shown in Figure 3. The groups represented by the band positions are shown in Table 1 [31]. It can be seen from the figure, there are obvious absorption peaks at 1120 cm^−1^ and 1030 cm^−1^ on BN-KH550 compared with BN fillers without surface modification. Before FT-IR test, BN filler has been washed with ethanol and deionized water to ensure that there is no residual silane coupling agent in the test sample. Therefore, according to Table 1, the new absorption peak should be the stretching vibration peak of Si-O bond introduced by silane coupling agent, which shows that the surface modification has been successfully carried out on the surface of BN.

### 3.3. Mechanical Properties of BN/AgNWs/EP Composites

**Figure 4:**
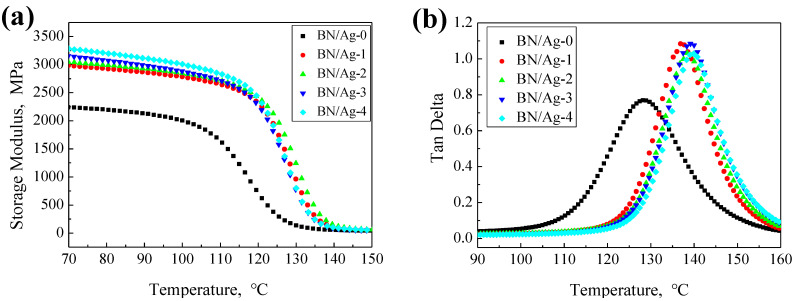
(**a**) Storage modulus of Composites (**b**) Loss tangent of Composites.

Figure 4a illustrates the storage modulus of the composites, which shows an obvious difference between the composites with and without AgNWs. Composites with AgNWs have a higher storage modulus than the other one. The composite fillers form a network structure in the resin matrix, which can help the epoxy resin better absorb and store energy at low temperature, so that the composite will not disperse and deform under external force. After entering the high-temperature range, the storage modulus of the composite decreases sharply. AgNWs in the filler enhance the internal interaction of the composite filler and improve the physical crosslinking of the composite. Therefore, the storage modulus of composite with composite filler is higher than that of composite with only BN filler. On the other hand, the AgNWs filler provides the stress conduction path during the fracture process, which improves the fracture toughness of the composite. Therefore, the storage modulus of the composites with composite fillers is higher than that of the composites with only BN fillers [32,33].

Figure 4b shows a symmetrical loss tangent curve, which indicates that the composites have cured completely. The peak of the curve represents the glass transition temperature. Composites with the composite fillers have a higher glass transition temperature, and, as the AgNWs content in composite fillers increases, so does the glass transition temperature. The AgNWs make the network of composite fillers much tighter, hindering the movement of resin segments in the epoxy matrix, resulting in the rise of the glass transition temperature.

**Figure 5:**
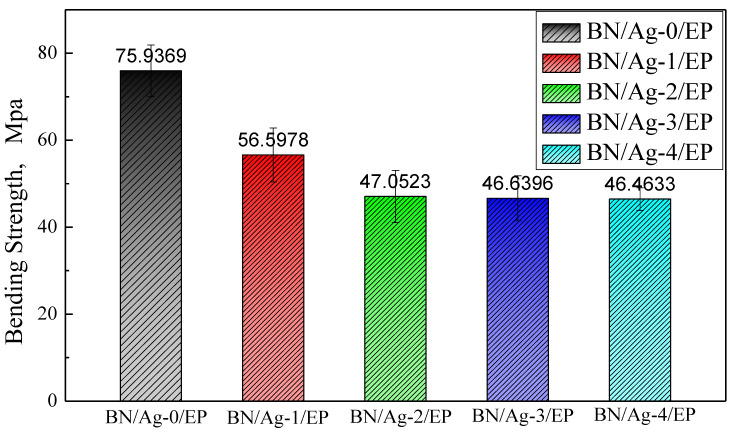
Bending strength of BN/AgNWs/EP composites.

The bending strength of the BN/AgNWs/EP composites with different filler mass ratios is shown in Figure 5. Here, one could see that, as the content of AgNWs in the composite fillers increased, the bending strength of the composites declined. Combined with Table 2, it can be seen that the addition of BN will cause the mechanical properties of BN/EP to be lower than that of EP, while coupling agent and DA can improve the interfacial compatibility and improve the mechanical properties of composites. Based on the principle of hydrolyzation by KH550, which brings the amino group to the surface of BN, the interface compatibility between BN and epoxy resin improves. Similarly, under PDA coating, AgNWs and epoxy have better interface compatibility. Therefore, the downward trend of bending strength becomes weaker. However, due to their high aspect ratio, AgNWs are more prone to breakage under external force. As a result of the destruction of the integral structure of the epoxy resin matrix, the matrix develops an increased number of defect sites. A composite with a higher content of AgNWs in the composite filler will therefore have a weaker bending strength.

### 3.4. Thermo Gravimetric Properties of BN/AgNWs/EP Composites

**Figure 6:**
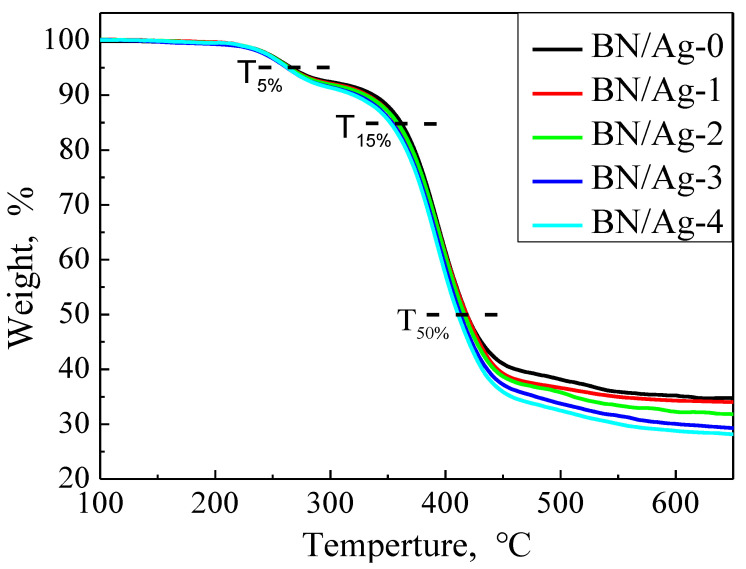
TGA curve of BN/AgNWs/EP Composites.

The thermal stability of the composites was studied by TGA from 100 °C to 700 °C at a heating rate of 10 °C/min under air. The results were shown in Figure 6. The T_5%_, T_10%_, and T_50%_ temperatures of the composites were shown in Table 3. The more the content of AgNWs in the composite fillers, the lower the thermal decomposition temperature of the composites at the same stage. Simultaneously, composites with fillers containing more AgNWs have a lower thermal decomposition temperature than those without. AgNWs in composite filler make the network of the filler more complete and have a broader influence range. AgNWs are long enough to reach all corners of the material, which results in faster heat transfer and faster decomposition of the composite.

### 3.5. Thermal Conductivity of BN/AgNWs/EP Composites

**Figure 7:**
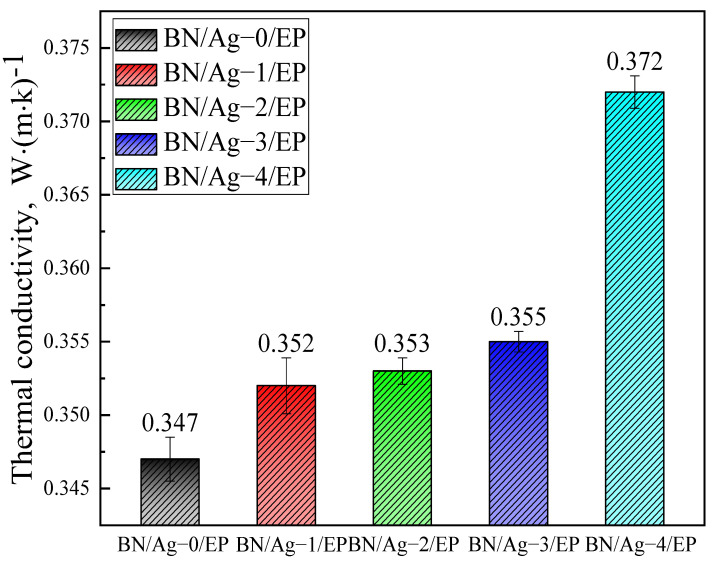
Thermal conductivity of BN/AgNWs/EP Composites.

The volume content of the BN fillers in the composites is 20 vol%. As illustrated in Figure 7, A higher amount of AgNWs is associated with a higher thermal conductivity, because AgNWs have a much higher thermal conductivity than epoxy resins. In addition, Table 4 shows the comparison of thermal conductivity data of BN/EP composites between others and this study. Combined with Table 4, it can be found that the addition of BN can improve the thermal conductivity of the composite. When AgNWs are not added, the BN nanosheets inside the matrix are not connected; thus, heat moves very slowly between the BN nanosheets and can escape easily. After adding AgNWs, which connects the BN nanosheets separated from each other in the thermal conduction network like the “bridge”, heat moves along the AgNWs between the BN nanosheets, increasing the speed and directivity of heat transfer. Besides, the addition of AgNWs completes the thermal network of fillers in the matrix. From the figure, it can be seen that the thermal conductivity of the composite has been raised 9%, which is not a significant improvement. This is because AgNWs is added in a small amount and can only be used as the thermal conduction “bridge” in the matrix without letting its high thermal conductivity play a decisive role [36,37,38,39,40,41].

**Figure 8:**
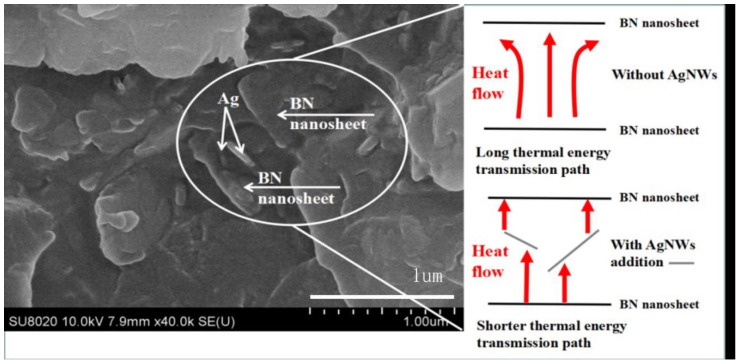
Thermal conduction mechanism of BN/AgNWs/EP composites.

Figure 8 illustrates the heat conduction mechanism of the composites. AgNWs are visible between BN nanosheets in the figure. AgNWs have a much higher thermal conductivity than EP matrix, so heat transfer between BN nanosheets will preferentially move along AgNWs. AgNWs act as a “bridge” in heat conduction and prevent disordered dispersion of heat in the matrix, while reducing the time and span of heat transfer between BN, and improving the thermal conductivity of composite materials [42,43,44,45].

### 3.6. Dielectric Properties of BN/AgNWs/EP Composites

**Figure 9:**
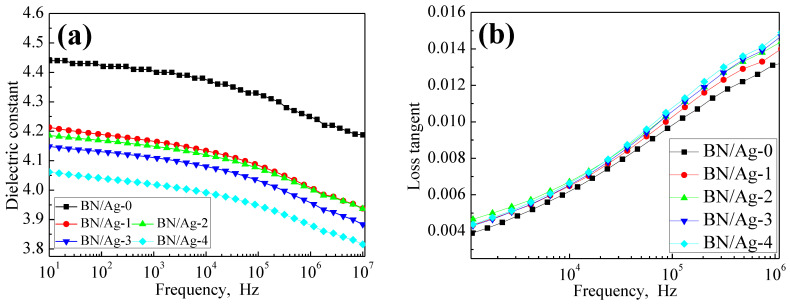
(**a**) Dielectric constant of Composites (**b**) Dielectric loss of Composites.

The dielectric properties of the composite were further compared by testing the dielectric constant and dielectric loss, and the results are plotted in Figure 9a,b. It can be seen from the figure that the dielectric constant of the composite decreases with the increase of AgNWs content. When the mass ratio of AgNWs to BN fillers increases from 0/300 to 4/300, the composites’ dielectric constant decreases slightly. At low frequency, the decrease of dielectric constant of the composite is less than 0.5 AgNWs restricts the movement of epoxy segments, causing the crosslinking degree of epoxy resin to increase gradually, thereby lowering the dielectric constant of the composite. The gaps inside the matrix caused by the poor contact between AgNWs and the resin matrix also make the dielectric constant of the composites decrease. However, the dielectric properties of the composite are still determined by the resin matrix and BN filler, because the content of AgNWs is too low, the polarization effect of AgNWs in the resin matrix is not obvious, and the dielectric cannot store more charges. Therefore, in general, the dielectric constant of the composite remains above 4.0 and still has well dielectric properties.

The dielectric loss of the composites with composite fillers increased slightly, which may be due to the defects in the resin matrix caused by AgNWs. However, the content of AgNWs is too small, so the crosslinking degree of the epoxy system in the composites does not change much. Overall, it is still the epoxy resin matrix itself that plays a significant role in the loss of the composites.

**Figure 10:**
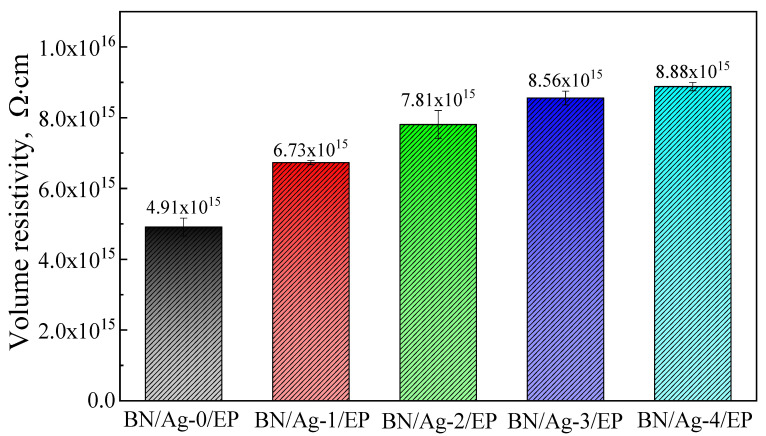
Volume resistivity of BN/AgNWs/EP Composites.

As shown in Figure 10, when the volume content of BN filler in the composite is 20 vol%, the volume resistivity of the composite increases gradually with the rise of AgNWs content in the mixed filler. The volume resistivity of BN/Ag-4/EP increased by 179.2% compared to BN/Ag-0/EP. Although AgNWs have high electrical conductivity, it can easily oxidize and form a layer of Ag_2_O on the surface. Similar to graphene oxide, the Ag_2_O formed on the surface of the silver wire filler also has good insulation properties. Increasing the amount of AgNWs in the composite filler also increases the amount of Ag_2_O on its surface. Thus, the resistance of the composite material to current in a unit volume is enhanced, and the volume resistivity of the composites is increased, and the insulation properties of the composite material are improved.

## 4. Conclusions

Taking EP as the matrix, by adjusting the content of agnws in the composite filler, it is known that the thermal conductivity of BN/agnws/EP composites increases with the increase of AgNWs content. Because BN has extremely high thermal conductivity, AgNWs are added into BN as mixed filler, BN is used as “point” and AgNWs are used as “bridge”, BN filler is connected, and a thermal conductivity network is constructed between EP substrates.

BN and AgNWs fillers were surface treated to improve the compatibility between fillers and matrix. Therefore, the change of composite filler ratio has little effect on the bending strength of the composite.

With the increase of AgNWs content, the dielectric constant of the composite decreases by 0.38 at low frequency, and the dielectric loss changes little. This is because AgNWs limits the movement of EP chain segments, resulting in the gradual increase of EP crosslinking degree. However, the content of AgNWs is not high, so it has little effect on the dielectric constant and dielectric loss of the composite.

With the increase of AgNWs content, the volume resistivity of the composites increased by 179.2%, and the insulation properties of the composites were improved. This is due to the high conductivity of AgNWs and its oxidized Ag_2_O.

In general, the composite filler with the mass ratio of AgNWs and BN of 4/300 can make the composite have better properties.

## Figures and Tables

**Table 1:** Group and band position of BN infrared spectrum.

Group	Band Position (cm^−1^)	Pure BN	BN-550	Vibration Mode
Si-O	1030, 1120	No	Yes	Telescopic vibration
h-BN sp^2^	804	Yes	Yes	Bending vibration
h-BN sp^2^	1380	Yes	Yes	Telescopic vibration

**Table 2:** Comparison of mechanical properties of composites with experimental data of others.

Case 1 [34]	Tensile Strength	Case 2 [35]	Tensile Strength	This Research	Bending Strength
BN/EP	14.6	EP	46.1	BN/EP	75.94
KH550/BN/EP	15.1	BN/EP	35.9	BN/Ag-1/EP	56.60
KH550-BN/EP	14.8	DA-BN/EP	58.7	BN/Ag-4/EP	46.46

**Table 3:** T_5%_, T_10%_ and T_50%_ temperatures of BN/AgNWs/EP Composites.

Samples	T_5%_ (°C)	T_10%_ (°C)	T_50%_ (°C)
BN/Ag-0/EP	2.66 ± 0.022 × 10^2^	3.38 ± 0.028 × 10^2^	4.19 ± 0.005 × 10^2^
BN/Ag-1/EP	2.65 ± 0.021 × 10^2^	3.32 ± 0.030 × 10^2^	4.19 ± 0.005 × 10^2^
BN/Ag-2/EP	2.64 ± 0.020 × 10^2^	3.31 ± 0.034 × 10^2^	4.17 ± 0.005 × 10^2^
BN/Ag-3/EP	2.62 ± 0.020 × 10^2^	3.23 ± 0.042 × 10^2^	4.13 ± 0.005 × 10^2^
BN/Ag-4/EP	2.73 ± 0.019 × 10^2^	3.21 ± 0.040 × 10^2^	4.11 ± 0.005 × 10^2^

**Table 4:** Comparison of thermal conductivity data with other experiments.

Case 1 [42]	Thermal Conductivity	Case 2 [43]	Thermal Conductivity	This Research	Thermal Conductivity
BN/EP	0.27	EP	0.21	BN/EP	0.347
ODA/BN/EP	0.32	1%-BN/EP	0.32	BN/Ag-1/EP	0.352
HBP/BN/EP	0.33	2%-BN/EP	0.36	BN/Ag-4/EP	0.374

## Data Availability

Not applicable.
